# Photosynthesis limitations in cacao leaves under different agroforestry systems in the Colombian Amazon

**DOI:** 10.1371/journal.pone.0206149

**Published:** 2018-11-01

**Authors:** Juan Carlos Suárez Salazar, Luz Marina Melgarejo, Fernando Casanoves, Julio A. Di Rienzo, Fabio M. DaMatta, Cristina Armas

**Affiliations:** 1 Universidad de la Amazonia, Facultad de Ingeniería, Programa de Ingeniería Agroecológica, Florencia-Caquetá, Colombia; 2 Universidad Nacional de Colombia - Sede Bogotá. Departamento de Biología, Laboratorio de Fisiología y Bioquímica Vegetal, Bogotá, Colombia; 3 CATIE (Centro Agronómico Tropical de Investigación y Enseñanza), Turrialba, Costa Rica; 4 Facultad de Ciencias Agropecuarias, Universidad Nacional de Córdoba, Córdoba, Argentina; 5 Departamento de Biologia Vegetal, Universidade Federal de Viçosa, Viçosa, Minas Gerais, Brasil; 6 Estación Experimental de Zonas Áridas, Spanish National Research Council (CSIC), La Cañada de San Urbano, Almería, Spain; National Research Council of Italy, ITALY

## Abstract

Cacao (*Theobroma cacao* L.) has traditionally been considered a crop that requires shade, and consequently it is implemented under agroforestry systems (AFs) in order to regulate the level of incident solar radiation. However, optimal shade levels for this tree crop may vary depending on the climate conditions of where it is grown. Here we analyzed the physiological performance of cacao under three different AFs in the Colombian Amazon that differed in solar radiation patterns: high *(H*_*PAR*_*)*, medium *(M*_*PAR*_*)*, or low *(L*_*PAR*_*)* mean daily incident radiation. The physiological performance was characterized using photosynthetic variables in leaves such as light- and CO_2_-response curves, chlorophyll *a* fluorescence parameters, and total chlorophyll and carotenoid contents, in conjunction with other leaf functional traits. Cacao trees exposed to *H*_*PAR*_ showed an improved physiological performance as compared to those from the other two AFs. Compared to *M*_*PAR*_ and *L*_*PAR*_, cacao trees in *H*_*PAR*_ doubled the rate of net carbon assimilation and reached higher maximum rates of RuBisCO carboxylation and RuBP regeneration. Moreover, cacao trees in *H*_*PAR*_ presented photoprotection mechanisms that avoided photoinhibition, which was accompanied by a greater non-photochemical quenching coefficient and biochemical and morphological adjustments (low chlorophyll but higher carotenoid contents and low specific leaf area) compared to cacao trees from the other AFs. Overall, our results show that, due to the high cloud cover in the Colombian Amazon, cacao plantations under conditions of sparse shade maximized their carbon use, showing an improved physiological performance as a result of higher photosynthetic rates and energy dissipation mechanisms. If the crop were managed with sparse shade, the paradigm that favors the cultivation of cacao under shade would be called into question in the Colombian Amazon and other regions with similar climatic conditions.

## Introduction

Cacao (*Theobroma cacao* L.), or cocoa, is one of the most important perennial crops in the world [1]. It is native to the Amazon rainforest [2], specifically the Colombian-Brazilian region straddling the Caquetá River [3], and has developed under high levels of precipitation (1,500–2,000 mm) and understory low light conditions [4]. The species shows photosynthetic characteristics of a shade tolerant species, exhibiting saturated rates of net carbon assimilation (*A*) at photosynthetically active radiation (*PAR*) levels between 200 and 750 μmol m^-2^ s^-1^ [5–7], as well as a low light compensation point (*LCP*) between 5 and 57 μmol m^-2^ s^-1^ [5, 7, 8] and a maximum *A* within the range of 1 and 8 μmol m^-2^ s^-1^ [5–7, 9]. For these reasons, cacao crops around the world are usually established in shady environments [10, 11].

Solar radiation and sunshine (daylight hours) vary greatly across the cacao-growing regions of Colombia [12]. For this reason, it is suggested that the structure (spatial distribution) and composition of cacao agroforestry systems (AFs) should be adjusted to generate an adequate level of shade for the cacao crop due to its sensitivity to high solar radiation inputs [11, 13, 14]. In parallel, the use of shelter trees that give shade to cacao plantations can modify the microclimatic conditions (e.g., temperature, humidity, vapor pressure deficit), which can potentially affect cacao’s stomatal conductance, transpiration rates, and *A* [15] as well as its agronomic performance [16].

Cloudy days prevail in the Colombian Amazon. Even on these days, average values of solar radiation of 766 μmol m^-2^ s^-1^, mean temperature of 22 °C, and humidity of 85% have been reported [12]. Due to these prevailing environmental conditions, there is some uncertainty on the real need to establish cacao plantations under shade conditions (AFs). In this regard, we previously evaluated cacao crops under AFs that are typical of the Colombian Amazon; we characterized the species composition, canopy structure and light availability in these AFs, which differ in degree of shade according to the shelter species that accompany the cacao plant, as well as the density of these species [17] (see Materials and methods). In this present study we analyzed the effect of transmitted radiation levels on the physiological performance of cacao plants by measuring chlorophyll (Chl) *a* fluorescence parameters and photosynthetic traits in leaves. We expected that the optimal physiological behavior of cacao would occur in the AFs with the greatest shade and that receive the least radiation due to a high density and diversity of accompanying species (the *Diversified multistrata* or *L*_*PAR*_ treatment, which receives a mean daily *PAR* transmission levels of 28%; *PAR* = 300 μmol m^-2^ s^-1^). These *PAR* values are within the optimal values for cacao crops, as reported by other authors [5, 6, 11]. Proper management of the cacao crop within AFs could translate into improvements in photosynthetic rates given that a decrease in solar radiation loads may improve the microclimatic conditions for the cacao plants, thus allowing greater leaf gas exchange rates and ultimately improving the cacao agronomic performance [14, 18, 19].

## Materials and methods

### Study area, agroforestry systems and sampling procedures

**Fig 1:**
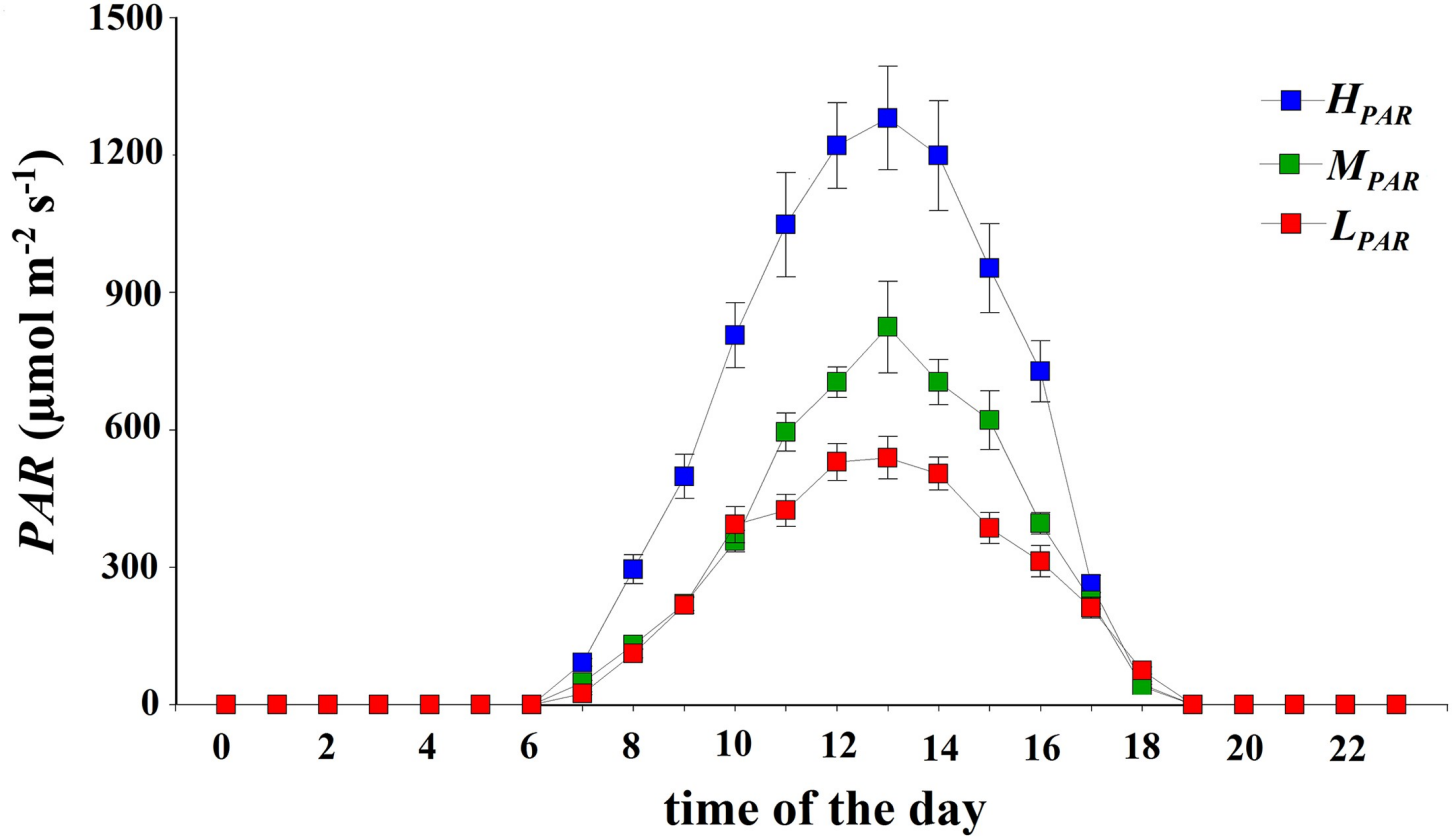
Daily curve of incident *PAR* in each agroforestry system. The agroforestry systems for cacao were: *cacaotal* with Musaceae plantation and high mean daily incident radiation (700 μmol m^-2^ s^-1^; *H*_*PAR*_), low diversity of clustered vegetation and medium mean daily incident radiation (400 μmol m^-2^ s^-1^; *M*_*PAR*_), and diversified multistrata vegetation and low mean daily incident radiation (300 μmol m^-2^ s^-1^; *L*_*PAR*_).

Measurements were taken for cacao plants under different AFs at the Macagual Center for Investigation—Amazon University (1°37’N and 75°36’W at 360 m a.s.l.), Colombia. The climate is warm-humid, characteristic of the ecosystem of tropical rainforests, with a mean annual precipitation of 3,800 mm, 1,200 sunshine hours year^-1^, a mean temperature of 25.5°C, and a relative humidity of 84%. Cacao plants were planted in October 2012 in a regular pattern with a distance among plants of 3 m in three plots or AFs of 25x50 m each, and with a total of 138 cacao plants per plot. The genotype used was the cacao clone CCN51 which was grafted onto plants of the IMC67 genotype, which was previously germinated in a nursery. After 5 months of grafting, the cacao saplings were planted in the experimental area. Each sapling was fertilized with one dose of 46 g of urea, 18 g of di-ammonium phosphate (DAP), and 279 g of K_2_SO_4_. All AFs plantations were established in a flat area with similar soil characteristics: soil had a clay-loamy texture with a bulk density that ranged between 1 and 1.3 g cm^-3^, a mean organic carbon content of 1.35%, mean pH of 5.5, available P content (Bray- II) of 2.58 mg kg^-1^, saturation of total bases of 7.1% (Ca: 0.38 meq 100g^-1^, Mg: 0.1 meq 100g^-1^, K: 0.14 meq 100g^-1^, Na: 0.1 meq 100g^-1^, total bases: 0.8 meq 100g^-1^), a cation exchange capacity of 11.3 cmol(-) kg^-1^, and an exchangeable aluminum content of 6.3 cmol(+) kg^-1^ with a percentage of Al saturation of 73.4%. Although the cacao plantation (*cacaotal*) was identical in the three plots, each AF differed from each other based on the type and density of the accompanying shelter species (henceforth called vegetation), that were planted in 2008 in rows with a North-South orientation and that created an upper canopy with varying levels of shade. Two of the AFs included accompanying timber species (*Cariniana pyriformis*, *Calycophyllum spruceanum*) and a third AF included Musaceae species (plantain; *Musa paradisiaca*). The three cacao AFs compared in this study were as follows: i. *cacaotal with Musaceae plantation and high mean daily incident radiation (H*_*PAR*_*)* with the plantain trees planted at a density of 527 trees ha^-1^ (i.e., one tree per 6 x 3 m area) allowing a daily mean transmitted *PAR* of 700 μmol m^-2^ s^-1^ (with mean midday values of 1,300 μmol m^-2^ s^-1^); ii. *low diversity of clustered vegetation and medium mean daily incident radiation (M*_*PAR*_*)* with the average shade generated by clusters of trees with thin crowns (*Cariniana pyriformis*, *Calycophyllum spruceanum*) allowing a daily mean transmitted *PAR* of 400 μmol m^-2^ s^-1^ (with mean midday values of 900 μmol m^-2^ s^-1^), and a density of 35 trees ha^-1^ (i.e., one tree per 12 x 25 m area); iii. *diversified multistrata vegetation and low mean daily incident radiation (L*_*PAR*_*)* with intense shade (*Cariniana pyriformis*, *Calycophyllum spruceanum*) allowing a daily mean transmitted *PAR* of 300 μmol m^-2^ s^-1^ (and mean midday values of 500 μmol m^-2^ s^-1^), and a density of 55 trees ha^-1^ (i.e., one tree per 12 x 15 m area). Macro-environmental factors (general climate and soil) remained constant for all plots. Due to experimental restrictions, the AFs were created in large plots and were not repeated. All of the measurements were performed in four cacao plants per AFs that were randomly chosen. Because of the clonal and coetaneous nature of the plants, it is assumed that differences recorded at the physiological level between systems are due to the effect of the AFs on the micro-environmental variables under the canopy, as well as to the effects of these variables on the physiological behavior of cacao, i.e. in the absence of confounding factors. The *PAR* was measured every 10 min with a quantum sensor (SQ-420, Apogee Instruments, Logan, UT, USA) located three meters high and installed with a WatchDog 2900ET weather station (Spectrum Technologies, Inc., Texas, USA) for each AF for 180 days; from here, we recorded the mean hourly *PAR* during daylight hours (Fig 1).

All of the measurements were performed in completely expanded healthy leaves (four leaves per plant) randomly selected from the middle layer of the cacao canopy; four plants per AFs were assessed.

### Photosynthetic light- and photosynthetic CO_2_-response curves in cacao under different AFs

Measurements were made using an infrared gas analyzer CIRAS-3 Portable Photosynthesis System (PP Systems Inc., Amesbury, MA, USA). The environmental conditions in the leaf cuvette consisted of a vapor pressure deficit (*VPD*) ranging from 1.0 to 1.5 kPa and a temperature of 25°C. Measurements were taken between 08:00 and 11:00 h (solar time), a partial concentration of CO_2_ of 400 ppm, and under artificial *PAR* provided by the LED light source of the cuvette.

The photosynthetic response curves to *PAR* intensity (henceforth, *A/PAR*) were generated by increasing *PAR* in 10 steps from 0 to 2,000 μmol m^-2^ s^-1^, a process that was carried out in a similar manner for the target cacao plants in each AF. Initially, the environmental conditions to which the leaves were exposed in the chamber were a *VPD* between 1.0 and 1.5 kPa, a leaf temperature of 25°C, and a partial concentration of CO_2_ of 50 ppm for 5 min to allow the stoma to open; subsequently *A/PAR* curves were obtained at a partial concentration of CO_2_ of 400 ppm. In order to determine the photosynthetic limitations of cacao that result from the microclimatic conditions in which it grows in each AF, the above data was used to determine different parameters derived from the *A/PAR* curves, such as light-saturated *A* (*A*_max_), light compensation point (*LCP*), dark respiration rates (*R*_d_), light saturation point (*LSP*), and apparent quantum efficiency (*Φ*_PAR_) determined from the slope of the initial linear portion of the *A/PAR* curve [20].

Photosynthetic response curves to CO_2_ concentration (henceforth, *A/C*i) were determined at a *PAR* of 500 μmol m^-2^ s^-1^ (based on the *A/PAR* curves), at 25°C and ambient O_2_ concentration following the recommendations of Long and Bernacchi [21]. Measurements were initiated at a partial concentration of CO_2_ of 400 ppm, which was gradually decreased to 50 ppm and subsequently increased in 15 steps up to 1,600 ppm of partial concentration of CO_2_ [22]. Leakage errors were corrected by measuring the CO_2_ response curves in dead leaves following the recommendations of Flexas et al. [23]. Different parameters derived from each *A/C*i curve were determined including maximum rate of RuBisCO carboxylation (*V*_cmax_), maximum rate of electron transport driving regeneration of RuBP (*J*_max_), and leaf respiration under light conditions (*R*_D_).

### Chl _*a*_ fluorescence parameters under the different AFs

Measurements were performed on the same leaves used to measure gas exchanges. This study used the chlorophyll fluorescence module (CFM-3) adapted for the infrared gas analyzer CIRAS-3 (PP Systems Inc., Amesbury, MA, USA). The CFM-3 provides chlorophyll fluorescence measurements using the pulse-amplitude modulation (PAM) technique. The maximum quantum yield of photosystem (PS) II (*F*_v_*/F*_m_) was determined at predawn (4:00 h; leaves in complete darkness) and by exposing the leaves to a saturating pulse of light (6,000 μmol m^-2^ s^-1^; 1s). After turning on a pulse of light, the leaf was exposed to a series of pulses of saturated light in order to obtain the maximum fluorescence yield in a light-adapted state (*F*_m_*′*).

The actual PSII quantum efficiency (*Φ*_PSII_), which measures the proportion of light absorbed by the PSII light-harvesting antenna that is used in the photochemical process [24], was determined in the light-adapted state as follows:

$$\Phi_{\text{PSII}=}\left(F_{\text{m}}\prime -F_{\text{s}}\right)/F_{\text{m}}\prime$$ (1)

where *F*_s_ is the fluorescence that was measured immediately before the application of pulses of light.

The apparent electron transport rate (*ETR*), which and is an indicator of overall photosynthetic capacity *in vivo*, was determined as follows [25]:

$$ETR=PAR\;\text{x}\;0,84\;\text{x}\;0,5\;\text{x}\;\Phi_{\text{PSII}}$$ (2)

The fraction of PSII centers which are in the open state according to the Lake model of the PSII photosynthetic unit (*q*_L_) was obtained as follows [25]:

$$q_{\text{L}}=\;qp\;x\;(F_{0}\prime /F_{\text{s}})$$ (3)

where *F*_0_*′* parameter is measured after an introduction of far-red illumination on light-adapted leaves (when all of the PSII reaction centers and electron acceptors are once again oxidized using a far-red light illumination).

Moreover, the non-photochemical quenching of *Chl*_a_ fluorescence (*NPQ*), which provides an indication of thermal energy dissipation through the PSII light-harvesting antenna (or heat dissipation of Chl excitation energy), was estimated as follows [25]:

$$NPQ=\left(F_{\text{m}}-\;F_{\text{m}}\prime \right)/F_{\text{m}}\prime$$ (4)

### Specific leaf area, total chlorophyll, and nitrogen

Specific leaf area (SLA) was determined using six leaf discs (3.14 cm^2^) exclude of the mid-vein for each leaf that had been previously used to measure gas exchange and Chl *a* fluorescence (n = 96 per AFs or 6 x 4 x 4 –six discs per leaf x four leaves per plant x four plants per AFs). The discs were dried to constant mass at 70°C, and the SLA was determined as the ratio between leaf disc area and its respective dry mass [26].

We followed the protocol described by Lichtenthaler [27] to determine leaf contents of total Chl (Chl_t_), Chl *a* and Chl *b*, and carotenoids for each leaf taking other six discs (3.14 cm^2^) from the same leaves that had been previously used to measure SLA. The Kjeldahl method [26] was used to determine total nitrogen.

### Data analysis

The Michaelis-Menten hyperbolic constant was used to adjust the *A/PAR* curves; the parameters *A*_max_, *LSP*, *LCP*, *R*_d_, and *Φ*_PAR_ were calculated following the equations described in Lobo et al. [28]. The model created by Farquhar et al. [29] (the ‘FvCB model’) was used to evaluate the *A/C*_i_ curve and estimate *V*_cmax_, *J*_max_, and *R*_D_ using the *plantecophys* package in R [30]. A generalized linear model (GLM) was adjusted for the different parameters derived from the *A/PAR* and *A/C*_i_ curves for each cacao plant in each AFs (fixed factor). The plant and leaf were included as random factors (n = 16). Likewise, a GLM was made for SLA, photosynthetic pigments, and nitrogen content, including the AF as the fixed factor. The plant, leaf and discs were included as random factors. The assumptions of normality and of homogeneity of variance were evaluated using an exploratory residual analysis. Differences between mean cacao plant responses in the AFs (fixed factor) were analyzed with the Fisher’s LSD post-hoc test at a significance of α = 0.05. Analyses were performed using the lme function in the nlme package [31] in R language software, version 3.4.4 [32], and using the interface in InfoStat [33].

## Results

The different mean levels of incident *PAR* in the three agroforestry systems had an effect on the photosynthetic response of cacao, its functional traits (e.g., SLA), and the chlorophyll and N content in leaves (Table 1).

**Table 1:** Photosynthetic and functional traits of cacao plants under different agroforestry systems. Parameters derived from photosynthetic light (*A/PAR*) and CO_2_ (*A/C*_i_) response curves, specific leaf area, and pigments under different cacao agroforestry systems (AFs): *cacaotal* with Musaceae plantation and *High* mean daily incident radiation (*H*_*PAR*_), low diversity of clustered vegetation and *Medium* mean daily incident radiation (*M*_*PAR*_), and diversified multistrata vegetation and *Low* mean daily incident radiation (*L*_*PAR*_). The results include means ± SE (n = 4).

			Cacao agroforestry systems	
Parameter	Unit	*H*_*PAR*_	*M*_*PAR*_	*L*_*PAR*_
*A*_max_	μmol m^-2^ s^-1^	8.1 ± 0.1^a^	5.9 ± 0.1^b^	3.3 ± 0.1^c^
	μmol kg^-1^ DM s^-1^	121 ± 2^a^	98 ± 1^b^	60 ± 1^c^
*R*_d_	μmol m^-2^ s^-1^	2.5 ± 0.3^a^	0.5 ± 0.2^b^	0.4 ± 0.1^b^
	μmol kg^-1^ DM s^-1^	46 ± 4^a^	8 ± 2^b^	6 ± 2^b^
*LSP*	μmol m^-2^ s^-1^	546 ± 54^a^	577 ± 63^a^	316 ± 73^b^
*LCP*	μmol m^-2^ s^-1^	15.2 ± 0.6^a^	14.5 ± 0.1^a^	14.7 ± 0.1^a^
Φ_PAR_ *	μmol CO_2_ μmol photons^-1^	3.1 ± 0.1^b^	3.2 ± 0.1^b^	3.8 ± 0.1^a^
*V*_cmax_	μmol CO_2_ m^-2^ s^-1^	24.9 ± 0.9^a^	19.6 ± 1.5^b^	17.8 ± 1.2^b^
*J*_max_	μmol CO_2_ m^-2^ s^-1^	48.1 ± 1.3^a^	36.1 ± 3.4^b^	46.2 ± 4.7^a^
*R*_D_	μmol CO_2_ m^-2^ s^-1^	1.6 ± 0.1^a^	0.9 ± 0.1^b^	1.0 ± 0.2^b^
*SLA*	m^2^ kg^-1^	14.94 ± 0.32^c^	16.83 ± 0.26^b^	18.59 ± 0.31^a^
Chl_t_	g kg^-1^ DM	1.33 ± 0.02^b^	1.17 ± 0.01^c^	2.04 ± 0.01^a^
Car	g kg^-1^ DM	0.41 ± 0.04^a^	0.31 ± 0.04^b^	0.30 ± 0.03^b^
Chl/Car		3.29 ± 0.03^c^	3.83 ± 0.02^b^	7.03 ± 0.15^a^
Chl *a*/*b*		2.79 ± 0.05^b^	3.17 ± 0.04^a^	1.28 ± 0.02^c^
Chl/N	mg kg^-1^ DM	1.99 ± 0.02^a^	1.30 ± 0.01^c^	1.66 ± 0.01^b^
N	g kg^-1^ DM	12.4 ± 0.3^c^	15.2 ± 0.2^b^	18.4 ± 0.5^a^
	g m^-2^ DM	0.67 ± 0.02^c^	0.90 ± 0.01^b^	1.23 ± 0.03^a^

*A*_max_: light-saturated net carbon assimilation rate, *R*_d_: dark respiration rate, *LSP*: light saturation point, *LCP*: light compensation point, *Φ*_PAR_: quantum efficiency, *V*_cmax_: Maximum carboxylation rate, *J*_max_: Maximum rate of regeneration of ribulose-1,5-bisphosphate controlled by electron transport, *R*_D_: Leaf respiration in light conditions, SLA: Specific leaf area, *Chl* t: Total chlorophyll, *Car*: Carotenoid, N: Nitrogen. DM: Dry mass.

*1×10^−3^.

^a, b, c^:Values in lines with different letters indicate significant differences between AFs (post-hoc LSD Fisher, p <0.05).

**Fig 2:**
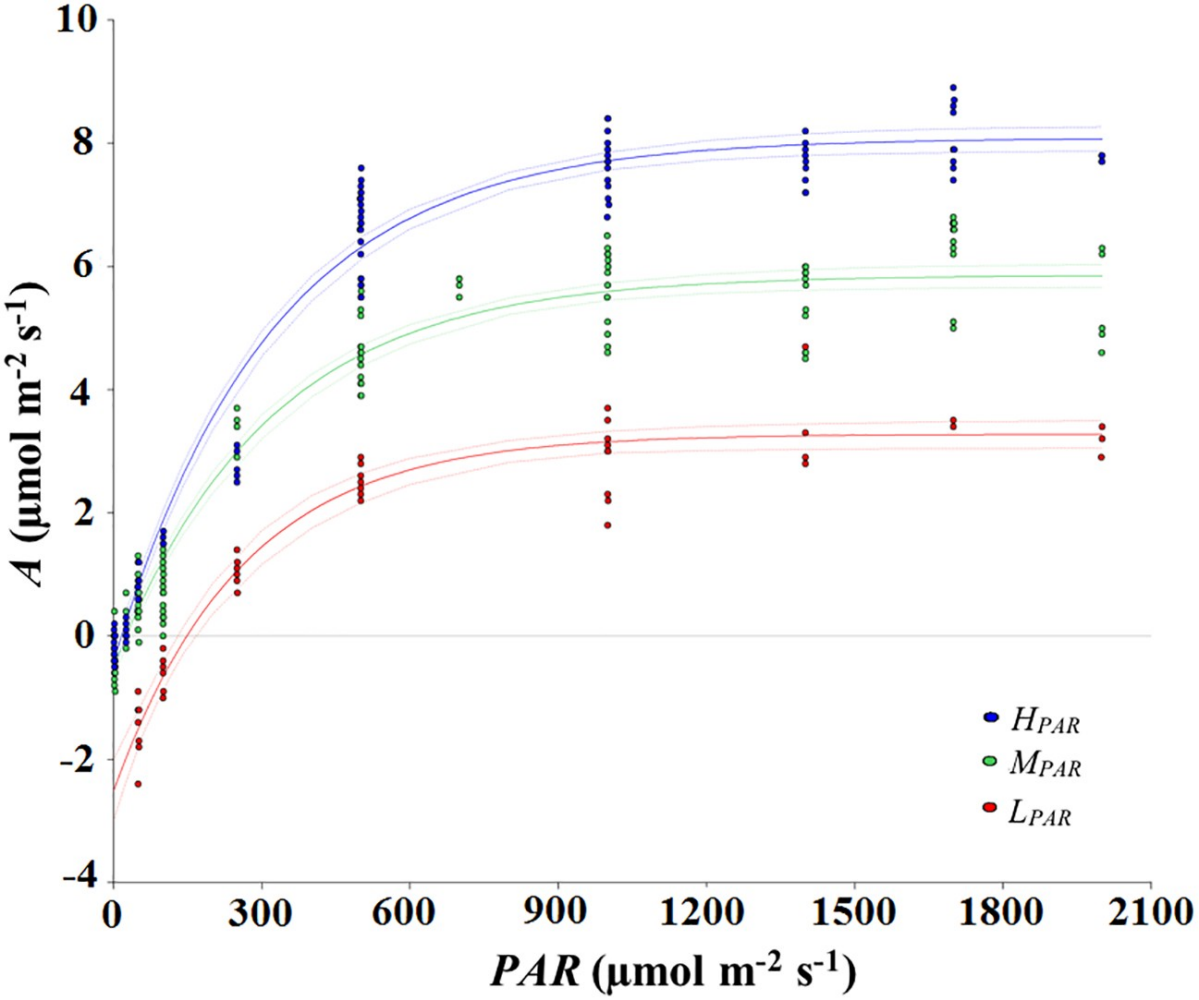
Leaf photosynthesis-*PAR* response curves. *A* is the net carbon assimilation rate. The agroforestry systems for cacao were: *cacaotal* with Musaceae plantation and high mean daily PAR incident radiation (700 μmol m^-2^ s^-1^; *H*_*PAR*_), low diversity of clustered vegetation and medium mean daily PAR (400 μmol m^-2^ s^-1^; *M*_*PAR*_), and diversified multistrata vegetation and low mean daily PAR (300 μmol m^-2^ s^-1^; *L*_*PAR*_).

**Fig 3:**
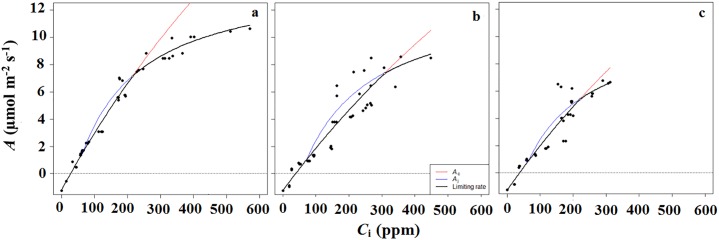
Leaf photosynthesis-CO_2_ response curves of cacao as modelled using the FvCB model. *A* is the net carbon assimilation rate, *C*_i_ is the intercellular CO_2_ concentration. Symbols represent the measured values, and the black line represents the fitted FvCB model of photosynthesis. Colored lines indicate the two photosynthesis rates in the FvCB model. *A*_c_ and *A*_j_ are the gross photosynthesis rates when RuBisCO activity and RuBP regeneration are limiting, respectively. **a.** *cacaotal* with Musaceae plantation and high mean daily *PAR* incident radiation (700 μmol m^-2^ s^-1^; *H*_*PAR*_), **b.** low diversity of clustered vegetation and medium mean daily *PAR* (400 μmol m^-2^ s^-1^; *M*_*PAR*_), **c.** diversified multistrata vegetation and low mean daily *PAR* (300 μmol m^-2^ s^-1^; *L*_*PAR*_).

The cacao plants exhibited the greatest photosynthetic efficiency (*A*_max_) in the AF with the highest amount of mean daily *PAR* (*H*_*PAR*_); cacao *A*_*max*_ in *H*_*PAR*_ was double the value recorded in *L*_*PAR*_ and higher than that recorded in *M*_*PAR*_ (Fig 2, Table 1). On the other hand, the leaves of the cacao plants in the system with the lowest amount of *PAR* (*L*_*PAR*_) exhibited the highest values *Φ*_PAR_ across AFs, whereas both *LCP* and *LSP* values were similar across AFs. The parameters derived from the *A/C*_i_ curves show that *V*c_max_ reached its maximum in the *H*_*PAR*_, whereas the maximum rate of electron transport driving regeneration of RuBP (*J*_max_) was similar among cacao plants growing in *H*_*PAR*_ and *L*_*PAR*_, but different from those growing in *M*_*PAR*_ (Fig 3. Table 1).

**Fig 4:**
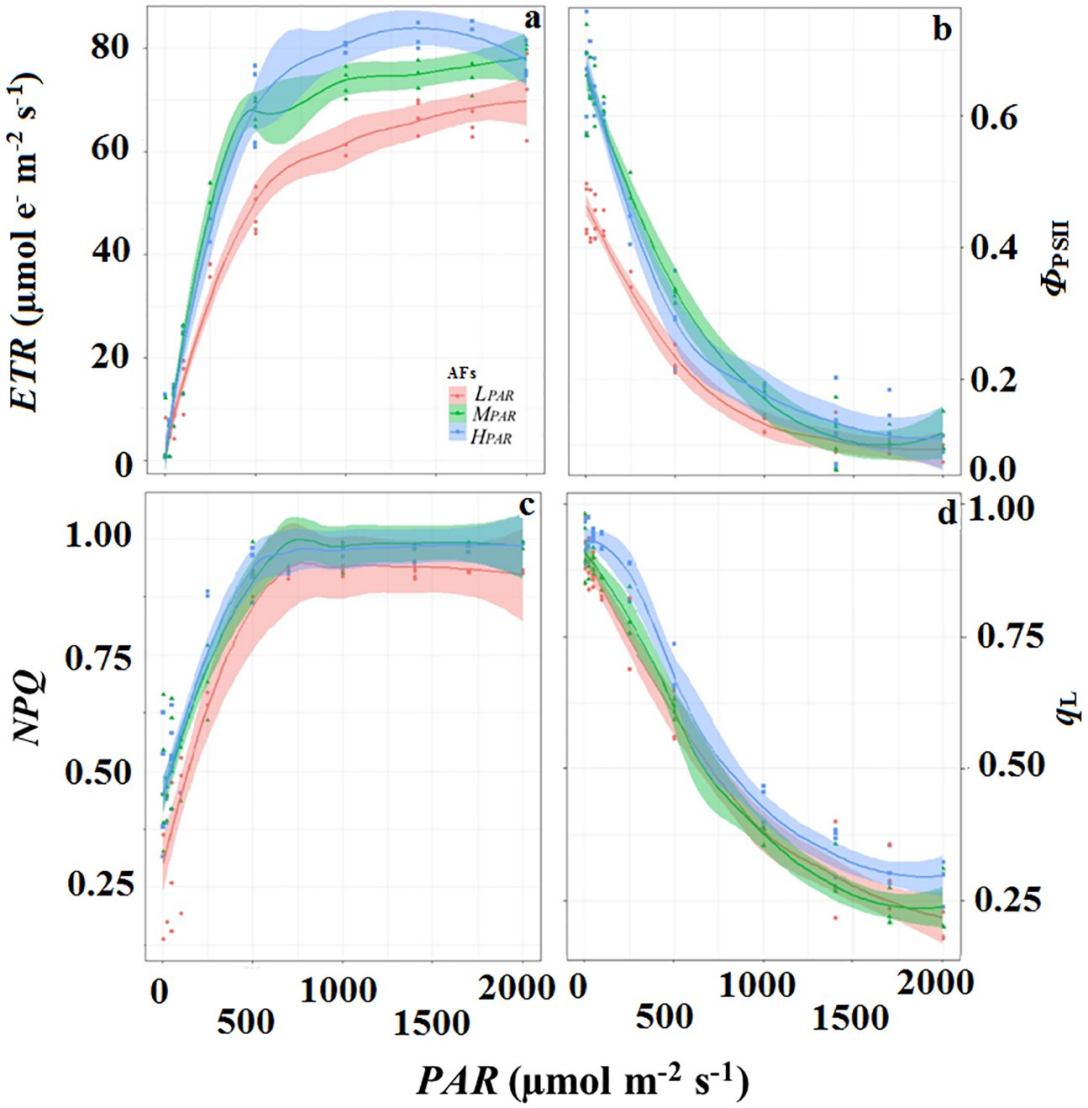
Chl _*a*_ fluorescence parameters as a function of *PAR* in cacao leaves. (a) electron transport rate (*ETR*); (b) actual PSII quantum yield (*Φ*_PSII_); (c) non-photochemical quenching (*NPQ*) coefficient; (d) photochemical quenching (*q*_*L*_) coefficient. Each agroforestry system is depicted by one curve (continuous line) in conjunction with confidence levels (shaded). Legend of the agroforestry systems is the same as in Figs 1 and 2.

The *F*_v_*/F*_m_ mean values were similar for all AFs (0.81±0.01), suggesting that cacao plants in our study did not exhibit photosynthesis photoinhibition [25], whereas other Chl fluorescence parameters differed significantly between AFs (Fig 4). The electron transport rate (*ETR*) was higher in cacao plants cultivated in *H*_*PAR*_ (8.5% and 18% higher compared to *M*_*PAR*_ and *L*_*PAR*_, respectively, at a radiation of 1,500 μmol m^-2^ s^-1^) and similar in the *M*_*PAR*_ and *L*_*PAR*_ systems (Fig 4a). The PSII quantum efficiency (*Φ*_PSII_; Fig 4b) and the photochemical quenching coefficient (*q*_L_; Fig 4d) gradually decreased as the intensity of radiation increased, with the minimum values for the cacao plants recorded in the *L*_*PAR*_ system. In the plants grown in the *H*_*PAR*_, the non-photochemical quenching (*NPQ;* Fig 4c) exhibited tendencies similar to those exhibited by the *ETR* results; however, more energy was dissipated in the form of heat—between 750 and 2,000 μmol m^-2^ s^-1^ of *PAR*.

The SLA of cacao plants was inversely proportional to the mean incident *PAR* level across the different AFs, with SLA being 24.4% smaller in *H*_*PAR*_ than in *L*_*PAR*_ (Table 1). Radiation levels in the AFs had an effect on the content of photosynthetic pigments in cacao leaves. Generally, the leaves from *L*_*PAR*_ exhibited higher leaf contents of Chl_t_ (34.8%), Chl/Car ratio (53.2%), and N (45.5%) as compared to their counterparts from *H*_*PAR*_; the values recorded for *M*_*PAR*_ were distinct, but fell in between those of the other two systems (Table 1). As for the leaf contents of Car and the Chl/N ratio, they were 34.4% and 19.6% higher, respectively, in *H*_*PAR*_ than in *L*_*PAR*_ and *M*_*PAR*_ cacao plants. Lastly, the Chl *a*/*b* ratio was higher in *M*_*PAR*_ cacao plants than in the other AFs, with plants in *L*_*PAR*_ exhibiting the lowest ratios (Table 1).

## Discussion

In contrast to the assumption that cacao plants grow better under shade conditions, we showed that it exhibits optimal acclimatization to conditions of relatively high solar radiation. The optimal physiological performance of plants from *H*_*PAR*_ is likely to have been associated with the high cloud cover that is typical of the Colombian Amazon, a region that displays and annual average of only 3–4 daily radiation hours [12]. Irrespective, we showed compelling evidence demonstrating the ability of cacao plants to acclimate to *H*_*PAR*_ conditions: i. overall, these plants displayed a decrease in Chl_t_ as well as an increase in Chl *a*/*b* ratio; in parallel *H*_*PAR*_ plants also showed a decrease in Chl/Car ratio and higher *NPQ* values, which favor the dissipation of excess energy in the form of heat [34–37]; ii. despite the high solar radiation the *H*_*PAR*_ plants did not show chronic photosynthesis photoinhibition, as denoted by the values of *F*_v_/*F*_m_ ratio above 0.80 [25]; iii. these plants displayed the highest *A*_max_ (even on a per leaf mass basis) and *V*_cmax_ as compared with the plants from the other AFs, suggesting higher RuBisCO activity [38]. Collectively, these acclimative responses to high solar radiation, in addition to improving the photosynthetic performance, should also contribute to better growth and crop yields in *H*_*PAR*_ than in *M*_*PAR*_ or *L*_*PAR*_ conditions.

In contrast to be *H*_*PAR*_ plants, their *L*_*PAR*_ counterparts showed high contents of photosynthetic pigments coupled with an increase in leaf N content and Chl/N ratio; these responses were probably related to adjustments in light harvesting ability. Both *L*_*PAR*_ and *M*_*PAR*_ plant showed a decrease in *R*_d_ and an increase in *Φ*_PSII_, which have also been reported for other species subjected to low irradiance and are considered as an adaptation strategy for survival under shade conditions. The increase in SLA is an additional strategy to cope with low *PAR*. In this regard, the greater SLA exhibited by cacao plants grown in the *L*_*PAR*_ might be considered as a mechanism to maximize photon capture efficiency, which may improve photosynthetic capacity [39] and carbon gain [40], in addition to concentrating resources invested in the construction of photosynthetic tissues [41]. This adaptation in plants subjected to low light intensities seems to be related to a decrease in leaf thickness and increase in leaf area for a more efficient absorption and optimization of photon capture [42]. However, as stated, these morphological (e.g., high SLA) and biochemical (increased leaf N content) adjustments of the highly shaded plants in *L*_*PAR*_ were not capable of improving its photosynthetic performance, as has also been observed in other studies conducted with different plant species under shady conditions [43].

Overall, many studies [5, 8, 11, 16, 35, 38] have demonstrated that cacao plants exhibit an optimal physiological behavior under conditions of low radiation, specifically at mean *PAR* levels of 400 μmol m^-2^ s^-1^, contrasting with what was found in our study. Results show that cacao plantations in *H*_*PAR*_ with mean radiation levels of ca. 700 μmol m^-2^ s^-1^ increased their *A*_max_ compared to plants from *M*_*PAR*_ or *L*_*PAR*_. Our results are in agreement with Jaimez et al. [44] who showed similar photosynthetic performance in cacao plants; other studies conducted in regions with high humidity and cloudy conditions point to similar results, such as Bently et al. [45] in the Amazonian Ecuador and de Araujo et al. [36] in the Atlantic coast of Brazil. Studies carried out under greenhouse conditions also demonstrate the adaptation of cacao to high radiation levels (1,200 μmol m^-2^ s^-1^) with *A* values of 5.7 μmol m^-2^ s^-1^ [9]. Taken together, these facts suggest that, under the conditions of the Colombian Amazon, the cacao plant has the capacity to acclimate to high light levels.

## Conclusions

This study shows that cacao plants growing under *H*_*PAR*_ exhibit improved carbon assimilation performance, demonstrating photosynthetic acclimatization to patterns of high solar radiation that resulted in higher *A*_max_ and *V*_cmax_ than in plants from the *M*_*PAR*_ or *L*_*PAR*_ systems. Even though the cacao plant is considered a demanding shade species, this study suggests that under the conditions of the Colombian Amazon, cacao plants can be even grown in monoculture instead of in AFs. Nonetheless, the high cloud cover conditions that prevail in the Colombian Amazon can differ from the other regions worldwide where cacao is grown; this could explain some discrepancies found in the literature as regarded to the optimal light conditions for the successful growth and production of cacao plants. Our results also suggests the need of case-by-case evaluation for the optimal conditions for cacao growth, taking into account the specific climatic conditions of a given geographic region. Given that cacao is one of the most important perennial crops in the world and its cultivation is a major economic resource in many developing regions, the provision of proper recommendations to the local producers is crucial for the sustainable implementation of agronomical practices resulting in improved crop yields.

## Supporting information

S1 File Leaf photosynthesis-CO_2_ response curves of cacao for *H*_*PAR*_. (TXT)

S2 File Leaf photosynthesis-CO_2_ response curves of cacao for *M*_*PAR*_. (TXT)

S3 File Leaf photosynthesis-CO_2_ response curves of cacao for *L*_*PAR*_. (TXT)

S4 File The photosynthetic response curves to *PAR* intensity for AFs. (TXT)

S5 File Chl _*a*_ fluorescence parameters as a function of *PAR* in cacao leaves for AFs. (TXT)
